# Oxygen therapy in early warning scores: a systematic review and meta-analysis

**DOI:** 10.1136/thorax-2024-222663

**Published:** 2025-05-13

**Authors:** Charlotte H Harrison, Phoebe Tupper, Stephen Gerry, Verena Michael, Jonathan P Bedford, Carolyn Smith, Chris Subbe, Oliver Redfern, Peter J Watkinson

**Affiliations:** 1 Critical Care Research Group, Nuffield Department of Clinical Neurosciences, University of Oxford, Oxford, UK; 2 Centre for Statistics in Medicine, Nuffield Department of Orthopaedics, Rheumatology and Musculoskeletal Sciences, University of Oxford, Oxford, UK; 3 Department of Critical Care, Milton Keynes University Hospital NHS Foundation Trust, Milton Keynes, UK; 4 Bodleian Health Care Libraries, University of Oxford, Oxford, UK; 5 Ysbyty Gwynedd, Bangor, Gwynedd, UK; 6 School of Medical Sciences, Bangor University, Bangor, UK; 7 Oxford Critical Care, Oxford University Hospitals NHS Foundation Trust, Oxford, UK; 8 Oxford Biomedical Research Centre, National Institute for Health and Care Research (NIHR), Oxford, Oxfordshire, UK

**Keywords:** Lung Physiology, Hypoxemia, Hypoxia, Critical Care

## Abstract

**Background:**

Early warning systems (EWS) used across the world typically assign a fixed number of points to patients receiving supplemental oxygen, regardless of amount. This ordinal binary approach may fail to recognise deteriorating patients who have an increasing oxygen requirement with otherwise stable observations. It is unclear whether weighting oxygen beyond binary scoring improves recognition of deterioration.

**Aims:**

We aimed to describe all general adult EWS that grade oxygen beyond binary scoring (part 1). Where reported, we summarised the performance of graded oxygen EWS in comparison to binary scoring (part 2).

**Methods:**

We systematically reviewed the literature, searching Embase, MEDLINE, CINAHL, Cochrane Central and Web of Science. We included studies of vital-sign-only EWS, for adult inpatients, which included grades of oxygen therapy above binary weighting (‘graded oxygen weighting’). We summarised methods of including graded oxygen therapy. We performed a random-effects meta-analysis of the effects of graded oxygen weighting inclusion in comparison to binary weighting. Risk of bias was assessed using the Prediction model Risk Of Bias ASsessment Tool.

**Results:**

15 studies reported the development of 16 EWS with graded oxygen weighting, classified by flow rate, delivery device and/or fraction of inspired oxygen. Four studies compared graded oxygen EWS to binary oxygen EWS. Meta-analysis showed a significant improvement in the performance of graded oxygen EWS over binary oxygen EWS (logit(AUROC)=0.19; 95% CI 0.094 to 0.285; p=0.002). 15/16 models were at high risk of bias.

**Conclusions:**

16 EWS with graded oxygen weighting were identified. Graded oxygen models had improved recognition of deterioration. Future work should explore the optimal method of oxygen classification and how this could be integrated into future EWS.

**PROSPERO registration number:**

CRD42024443362.

**WHAT IS ALREADY KNOWN ON THIS TOPIC**

- An early warning system (EWS) assigns points to patients’ vital signs to identify deterioration.
- Many EWS used internationally, such as the National Early Warning Score 2, include oxygen therapy as an ordinal binary variable; 0 points for room air, and a fixed number of points for supplemental oxygen.
- There have been concerns that this simplistic approach to scoring oxygen might delay recognition of deterioration, as patients can have an escalating oxygen requirement in the absence of other vital sign abnormalities.

**WHAT THIS STUDY ADDS**

- This is the first systematic review of EWS that quantify supplemental oxygen therapy beyond binary scoring.
- Our findings highlight the range of approaches to oxygen classification and potential biases introduced during model development and validation.
- Meta-analysis demonstrates that EWS with graded oxygen therapy have significantly better discrimination for detecting adverse patient outcomes than binary oxygen therapy EWS.

**HOW THIS STUDY MIGHT AFFECT RESEARCH, PRACTICE OR POLICY**

- This review provides objective evidence that recognition of deterioration would be enhanced with more detailed oxygen therapy scoring.
- The ‘best’ way of defining and scoring oxygen supplementation (both in terms of predictive performance and end-user functionality) requires further study.
- This work should be prioritised to ensure future EWS optimally use the supplemental oxygen therapy domain.

## Introduction

 Early warning systems (EWS) are widely used in hospitals to help detect patient deterioration.^1^ EWS assign points to vital sign measurements—the more abnormal the vital sign value, the greater the number of points.^2^ A score above a certain threshold should trigger a clinical review.^2 3^ EWS often score supplemental oxygen therapy in a binary manner—a patient breathing room air scores 0 points, and a patient requiring any supplemental oxygen scores a fixed number of additional points.

Perhaps the most commonly used EWS is the National Early Warning Score, in its second iteration (NEWS2).^3^ NEWS2 is mandated for use by NHS England across all acute hospitals,^3^ has been validated in over 40 studies^1^ and is used extensively around the world.^4 5^ Other nationally employed EWS, such as those in Ireland,^6^ New Zealand^7^ and Australia,^8^ also score oxygen therapy in a binary manner.

The binary approach to oxygen supplementation in EWS may limit recognition of the deteriorating patient.^9 10^ A deteriorating patient may have an escalating oxygen requirement with minimal changes to other vital signs, causing no change to their total score, and hence delayed recognition of deterioration.^11^ This limitation was highlighted during the COVID-19 pandemic in relation to NEWS2,^11^ but is relevant to all binary oxygen EWS. Since, there have been continued calls to develop the oxygen therapy component in EWS,^9^,^14^ including in guidance from the Thoracic Society of Australia and New Zealand.^15^

However, there is no consensus that quantifying oxygen therapy in greater detail does improve recognition of deterioration. Although prior examples exist,^9 16 17^ there is no agreed approach on how to handle oxygen within an EWS that prompts earlier recognition of deterioration, while maintaining simplicity for the user and standardisation across healthcare systems.

This systematic review describes published vital-sign-only EWS with oxygen graded beyond binary scoring and evaluates published differences in model performance between binary versus graded oxygen models. The term ‘graded oxygen weighting’ is used to refer to oxygen therapy weighted within a score in greater detail than a binary approach.

### Objectives

We conducted a systematic review to answer the following questions:

- Part 1: Which EWS have been developed that weight oxygen therapy beyond binary scoring (graded oxygen weighting)?
- Part 2: Does graded weighting of oxygen therapy in an EWS improve predictive performance, compared with binary scoring?

## Methods

Our systematic review protocol was registered on PROSPERO (CRD42024443362) and carried out in accordance with The Preferred Reporting Items for Systematic reviews and Meta-Analyses (PRISMA) statement^18^ (online supplemental appendices A and B).

### Eligibility criteria

#### Study type

We included primary research articles describing the development or adaptation of a vital sign only EWS with graded oxygen weighting (part 1), and/or validated the performance of a graded oxygen EWS compared with a binary oxygen EWS (part 2). We excluded reviews, editorials, comments, letters, case studies, trial protocols, conference abstracts, book chapters, non-peer reviewed preprints and animal studies.

#### Setting

The eligible population were adults admitted to hospital. EWS developed for use in the outpatient setting (including unselected emergency department populations and prehospital use), solely within an intensive care setting, paediatric populations or obstetric/maternity populations were excluded.

#### Part 1

Studies must have developed or adapted a vital sign only EWS to include graded oxygen weighting. An early warning score was defined as any model using two or more vital signs (in addition to oxygen therapy) to identify deteriorating patients.^1^ Vital signs included heart rate, respiratory rate, blood pressure, temperature, consciousness, urine output and oxygen saturation. Scores containing non-vital sign parameters (eg, age, laboratory values) were excluded. Oxygen therapy was required to be quantified in a manner beyond binary scoring. Binary scoring was defined as one level attributed to air and the other to any oxygen supplementation (as per the NEWS).^3^ Studies that only validated (without developing or altering) an oxygen graded EWS were excluded. The outcome was any measure of patient deterioration, such as (but not limited to) intensive care unit (ICU) admission, cardiac arrest and death.

#### Part 2

Studies must have validated the predictive performance of a vital sign only EWS with oxygen grading compared with an otherwise identical EWS with binary oxygen, at predicting deterioration among an adult inpatient population.

See online supplemental appendix C for a schematic of inclusion criteria.

### Search strategy

We searched the electronic bibliographic databases Embase and MEDLINE (on OVID) and CINAHL, Cochrane Central and Web of Science on their websites, for literature from 1946 to present. The search strategy and permalink for MEDLINE, based on concepts of ‘early warning scores’ and ‘oxygen’, are available in online supplemental appendix D. These search terms were adapted for use with the other bibliographic databases in combination with database-specific filters for controlled trials, where available. The initial searches were performed on 26 May 2023 and updated on 25 March 2024. Additional papers were sought through forward and backward citation tracking of included studies using Web of Science. Reference lists were examined at the point of inclusion in the study, and citation alerts were followed until 25 April 2024. We also examined reference lists of relevant systematic and narrative reviews.

In the event that a relevant oxygen graded EWS was mentioned in an otherwise ineligible study (eg, performing an external validation only), we performed backward citation tracking to identify the original model development study, or the first time it was described in the literature.

### Study selection and data extraction

All references were downloaded into the systematic review software Covidence (https://www.covidence.org/). After deduplication of studies, study screening (title and abstract), full text review, study selection and data extraction were performed by two independent reviewers. Disagreements were resolved with discussion and recourse to the original data. Items for data extraction were taken from the Critical Appraisal and Data extraction for Systematic Reviews of Prediction Modelling Studies checklist.^19^ A full list of data extracted can be found in the supplementary material (online supplemental appendix E).

### Risk-of-bias assessment

Two independent reviewers completed a quality assessment of included studies using the Prediction model Risk Of Bias ASsessment Tool (PROBAST).^20^ Discrepancies were resolved by discussion and recourse to the original data. Applicability was not assessed, as only studies meeting review inclusion criteria were considered. Studies eligible for part 1 were assessed according to the ‘development’ criteria of PROBAST, and studies eligible for part 2 were assessed according to the ‘validation’ criteria of PROBAST.

### Analysis

For part 1, analysis was descriptive, with results summarised using descriptive statistics, graphs, tables and narrative summary.

For part 2, we compared graded oxygen models to their binary oxygen equivalent, which represents the incremental predictive ability of graded oxygen.^21^ We therefore looked at the difference in area under the receiver operating characteristic (AUROC), with weighing by the square root of the validation sample size (number of observations). This has the added benefit of not needing to rely on deriving standard errors from CIs that were calculated using different approaches, or not reported with sufficient precision, and in some cases missing altogether.^21^ Hence, by using an approach different from the typical inverse variance weighting, it was not possible to obtain a formal estimate of heterogeneity. We used the logit(AUROC) due to the bounded nature of the AUROC. These established methods are demonstrated by Christodoulou *et al*.^22^

We prespecified subgroup meta-analyses where three or more studies reported a comparison of model performance in the same subgroup. We also performed a sensitivity analysis weighting studies by the square root of the validation sample size (patients/patient admissions). Analysis was performed in R software, using packages nlme, meta and metamisc.

## Results

**Figure 1:**
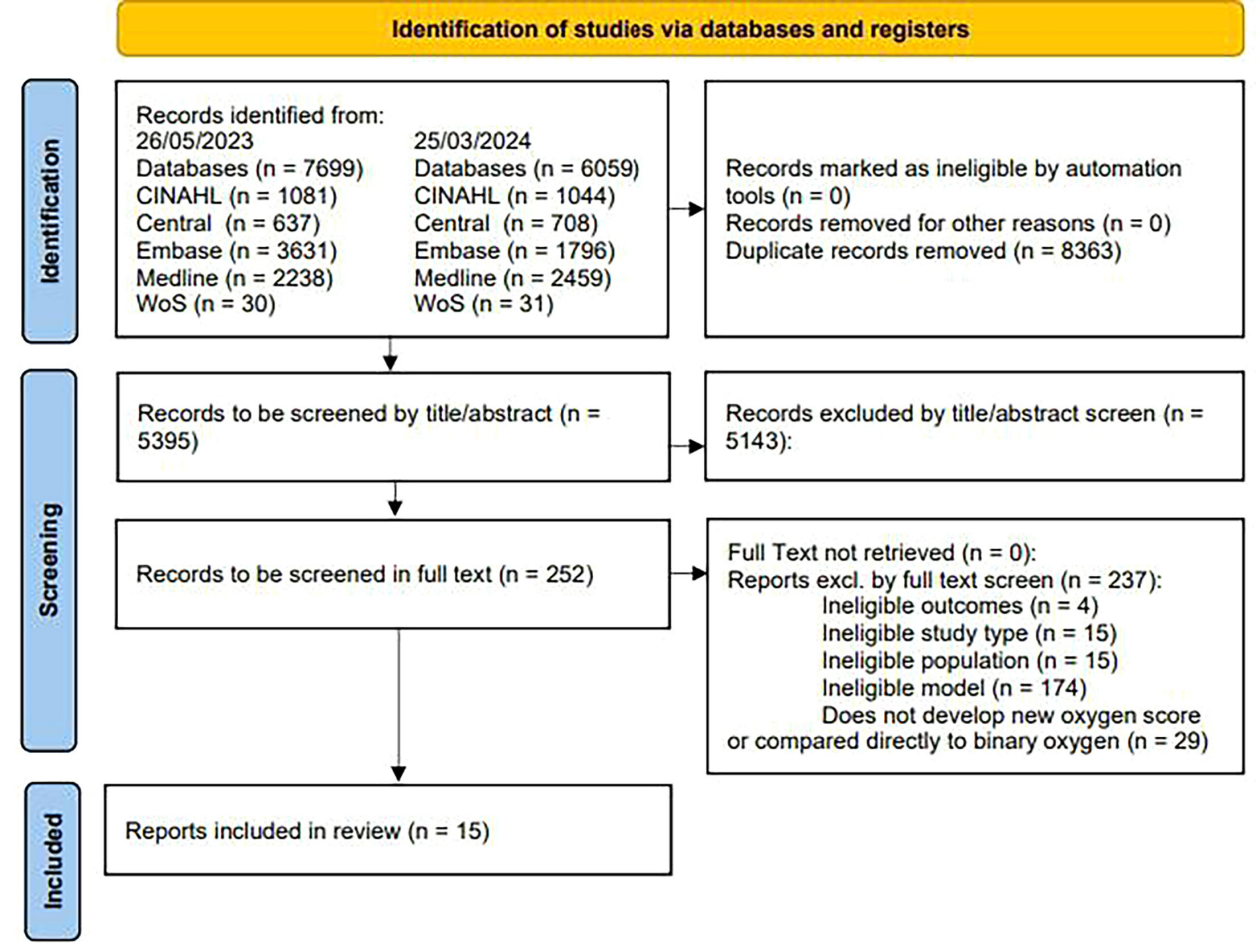
Flow chart showing the process of study identification for inclusion.

Of the 5395 non-duplicated studies screened, 252 underwent full-text assessment, with 15 studies included. These 15 studies described the development of 16 vital-sign EWS with graded oxygen weighting (part 1) and in 4 studies, the predictive performance of a graded oxygen model was compared with a binary oxygen EWS (part 2). The study selection process is summarised in the PRISMA flow diagram (figure 1).

### Part 1

15 studies reported the development of 16 vital-sign-only EWS with graded oxygen. The 16 models were: the Queensland Adult Deterioration Detection System (Q-ADDS),^23^ NEWS2+oxygen flow rate (3-day model),^24^ Logistic EWS,^16^ NEWS-fraction of inspired oxygen (FiO_2_) (predictive model),^10^ Nottingham EWS,^25^ Additive NEWS-FiO_2_ Score,^26^ Dynamic Early Warning Score (DEWS),^17^ the Quick COVID-19 Severity Index,^27^ the Modified Early Warning Score plus peripheral oxygen saturation (SpO_2_)/FiO_2_ score (MEWS_SF),^28^ NEWS-FiO_2_,^9^ the Novelty score,^11^ Leeds EWS,^29^ Hamilton Early Warning Score (HEWS),^30^ Early Warning Score.O_2_ (EWS.O_2_),^31^ Truncated EWS.O_2_^31^ and the Dynamic prediction model (Dynamic Individual Vital Sign Trajectory Early Warning Score (DyniEWS)).^13^ Viglino *et al* developed two scores.^31^

#### Model development

All studies employed a retrospective design. The most common method of model development was clinical consensus (7/16), followed by statistical modelling (6/16) and machine learning (1/16), or a combination of approaches (2/16). The median number of non-oxygen predictors in the model was 6. Online supplemental table F1 summarises key features of model development.

Most models used a simple additive approach, adding to an existing ‘base’ EWS, such as: Q-ADDS,^23^ NEWS2+oxygen flow rate (3-day model),^24^ the NEWS-FiO_2_ (predictive model),^10^ Nottingham EWS,^25^ MEWS_SF,^28^ NEWS-FiO_2_,^9^ the Leeds EWS^29^ and HEWS.^30^ DEWS,^17^ DyniEWS^13^ and the additive NEWS-FiO_2_ Score^26^ incorporated dynamic changes in observation trends.

Internal validation was performed in 7/16 models, specifically bootstrapping (3/16), temporal validation (3/16) and 10-fold cross validation (1/16). 13/16 models used a complete case analysis. Other strategies to manage missingness included K-nearest neighbour imputation,^24^ median value imputation^27^ and mean imputation.^11^

#### Model development cohort

Models were developed on a range of data sources, including Guy’s and St Thomas’ NHS Foundation Trust^10^; Nottingham University Hospitals^17 25 26^; King’s College Hospital and Princess Royal University Hospital, James Cook University Hospital, New Cross Hospital, Royal Papworth Hospital, University Hospitals Coventry and Warwickshire^13 16^; Asan Medical Centre, South Korea^28^; Oxford University Hospitals NHS Foundation Trust^9 11^; Grenoble Alpes University Hospital, France^31^; and Hamilton Health Sciences Electronic Medical Records, Canada.^30^ For two models, it was not clear which data sources were used.^27 29^ Online supplemental table F2 describes the model development cohorts.

Most models were developed on a respiratory population, such as COVID-19 patients,^10 24 27^ patients with viral pneumonia,^11^ patients admitted to a respiratory ward^17 25 26^ or admitted from the emergency department with dyspnoea.^31^ Other specialist non-respiratory populations were used, such as patients postcardiac surgery^13 16^ and patients admitted to a haematology oncology ward.^28^ NEWS-FiO_2_,^9^ HEWS^30^ and the Leeds EWS^29^ were developed in general adult admissions. Leeds EWS^29^ and MEWS_SF^28^ had additional inclusion criteria of patients requiring escalation to an outreach team/medical emergency team.

The median sample size for model development was 5417 patient admissions (range 220–224 912). 13/16 models used a composite primary outcome, usually of cardiac arrest, unplanned ICU admission and in-hospital mortality; 4 incorporated a respiratory event within the composite outcome, such as initiation of non-invasive ventilation (NIV), continuous positive airway pressure (CPAP) and/or high-flow nasal oxygen (HFNO).^11 27 31^ The median percentage of patient admissions experiencing the primary outcome was 12.3% (range 3.9%–46.8%).

#### Quality assessment for model development

We rated 15/16 models at high risk of bias according to the PROBAST development checklist.^20^ This was most commonly due to a ‘high’ rating in the analysis domain, because of complete case analysis and/or lack of internal validation. The NEWS2+oxygen flow rate^24^ was the only model at low risk of bias. The number of models at risk of bias in each individual domain is highlighted in table 1. Risk-of-bias assessments for each model are found in online supplemental table F3.

**Table 1:** Part 1, risk-of-bias assessment for model development

Rating	Participants	Predictors	Outcome	Analysis	Overall
High	5	1	3	15	15
Low	11	15	12	1	1
Unclear	0	0	1	0	0

#### Supplemental oxygen therapy

Table 2 summarises how oxygen was classified and used within each model. There were a range of units used to quantify oxygen, including: FiO_2_ (7/16), flow rate (3/16), delivery device (1/16), flow rate and FiO_2_ (3/16) and a combination of flow rate, FiO_2_ and delivery device (2/16).

**Table 2:** Part 1, summary of oxygen use in models

(A) Models with oxygen as a categorical variable								
Study	Oxygen model name	Method of oxygen classification	Category 1 range (value)	Category 2 range (value)	Category 3 range (value)	Category 4 range (value)	Category 5 range (value)	Category 6 range (value)
Forster *et al*^25^	Nottingham EWS	Flow rate	0–9 L/min (0)	10–14 L/min (1)	≥15 L/min (3)			
Haimovich *et al*^27^	qCSI	Flow rate	0–2 L/min (0)	3–4 L/min (4)	5–6 L/min (5)			
Lee *et al*^28^	MEWS_SF*	FiO_2_ (S/F ratio)	>316 (0)	236–315 (2)	≤235 (3)			
Tam *et al*^30^	HEWS	FiO_2_ or flow rate	Room air (0)	>0–5 L/min (1)	>5 L/min (3)			
				0.21–0.50 (1)	≥0.51 (3)			
Chiu *et al*^16^	Logistic EWS	Flow rate, FiO_2_ or delivery device	Room air (1)	>0–4 L/min (1.3)	≥5 L/min (2.13)	>0.45 (2.92)		
				0.25–0.34 (1.3)	0.35–0.44 (2.13)	Reservoir mask (2.92)		
Clarke *et al*^10^	NEWS-FiO_2_ (predictive model)	FiO_2_†	0.0–0.22 (0)	0.221–0.37 (1)	0.371–0.53 (2)	>0.53 (3)		
Forster *et al*^26^	Additive NEWS-FiO_2_ score‡	FiO_2_§	0.0–0.22	0.221–0.37	0.371–0.53	>0.53		
Malycha *et al*^9^	NEWS-FiO_2_	FiO_2_¶	0.0–0.22 (0)	0.221–0.37 (1)	0.371–0.53 (2)	>0.53 (3)		
Pittard^29^	Leeds EWS	Delivery device	Room air (0)	Oxygen therapy (1)	High flow (2)	BiPAP (3) CPAP(3)		
Zhu *et al*^13^	DyniEWS‡	Flow rate, FiO_2_ or delivery device	Room air	0.25–0.34	0.35–0.44	≥0.45 (3) Reservoir mask (3)		
				>0–4 L/min	≥5 L/min			
Campbell *et al*^23^	Q-ADDS	FiO_2_ or flow rate	0.21 (0)	0.22–0.35 (1)	0.36–0.40 (2)	0.41–0.49 (3)	≥0.50 (4)	
			<1 L/min (0)	1–4 L/min (1)	5–7 L/min (2)	8–10 L/min (3)	>10 L/min (4)	
Gonem *et al*^17^	DEWS‡	FiO_2_ or flow rate	0.21	0.22–0.24	0.25–0.28	0.29–0.35	0.36–0.50	0.51–1.0
			0 L/min	0.5–2.5 L/min	3–4 L/min	5–9 L/min	10–14 L/min	≥15 L/min

(B) Models with oxygen as a continuous variable			
Study	Oxygen model name	Method of oxygen classification	Other
Carr *et al*^24^	NEWS2+oxygen flow rate	Flow rate	Flow rate, beta-coefficient not reported
Pimentel *et al*^11^	Novelty score	FiO_2_	Treated as a continuous variable and combined with individual vital sign abnormalities, whole set of vital signs given overall score and compared with the multidimensional distribution of normal
Viglino *et al*^31^	EWS.O_2_	FiO_2_	Oxygen flow rate converted to FiO_2_ using the Wettstein *et al*^34^ conversions. For patients with HR≥50: (RR/(SpO_2_/FiO_2_))×(HR/100). For patients with HR<50: (RR/(SpO_2_/FiO_2_))×(50/HR)
Viglino *et al*^31^	Truncated EWS.O_2_	FiO_2_	Oxygen flow rate converted to FiO_2_ using the Wettstein *et al*^34^ conversions. RR(SpO_2_/FiO_2_)

Units of oxygen are displayed as FiO_2_ (0.21–1.0) and/or oxygen flow rate.

* The MEWS_SF is reported as S/F ratio.

† Conversion using Fuentes and Chowdhury^33^ equation.

‡ The coefficients are not reported for DEWS,^17^ the additive NEWS-FiO_2_ Score^26^ or DyniEWS^13^ as these models further managed the oxygen to include some dynamic reflection of change over time.

§ Conversions taken from the Malycha *et al*^9^ paper (Bateman equation).

¶ Conversion using Bateman equation for nasal cannulae, simple masks, tracheostomy masks, nebuliser mask, nasal humidified oxygen. Prescribed value for fixed performance masks.

BiPAP, bilevel positive airway pressure; CPAP, continuous positive airway pressure; DyniEWS, Dynamic Individual Vital Sign Trajectory Early Warning Score; EWS, early warning system; EWS.O_2_, Early Warning Score.O_2_; FiO_2_, fraction of inspired oxygen; HEWS, Hamilton Early Warning Score; MEWS_SF, Modified Early Warning Score plus SpO2/FiO2 score; NEWS, National Early Warning Score; Q-ADDS, Queensland Adult Deterioration Detection System; qCSI, Quick COVID-19 Severity Index; RR, respiratory rate; S/F, SpO_2_:FiO_2_ ratio; SpO_2_, peripheral oxygen saturation.

##### Oxygen conversion

Of the seven models using FiO_2_, six detailed how FiO_2_ was estimated from flow rate. There were three different formulas described. The Bateman equation^32^ was most frequently used, in the NEWS-FiO_2_,^9^ additive NEWS-FiO_2_ score^26^ and the novelty scores.^11^ The NEWS-FiO_2_ (predictive model)^10^ used the conventional prediction model.^33^ EWS.O_2_ and truncated EWS.O_2_ used the conversions of Wettstein *et al*.^34^ Finally, in MEWS_SF,^28^ FiO_2_ was used within an SpO_2_/FiO_2_ (SF) ratio and categorised according to previous literature^35^; however, the method of calculating FiO_2_ was not stated.

##### Predictor handling of oxygen within the model

Weighting oxygen as a continuous variable occurred in 4/16 models: the NEWS+oxygen flow rate,^24^ the Novelty score,^11^ EWS.O_2_^31^ and the truncated EWS.O_2_^31^ (table 2b).

Weighting oxygen as a categorical variable occurred in 12/16 models. The median number of oxygen categories was 4. Different thresholds and points were assigned to each category (table 2a). An explanation of why thresholds were chosen and points/coefficients assigned was often not provided. In the NEWS-FiO_2_ model, this was achieved using decision tree analysis.^9^ Two subsequent models^10 26^ replicated the points and thresholds of the NEWS-FiO_2_ model.

A justification of how to handle oxygen within the model was often not provided (5/16), or resulted from statistical approaches (4/16), clinical consensus (3/16), previous literature (3/16) or machine learning (1/16).

### Part 2

#### Overview

Four different oxygen models were compared with their binary oxygen equivalent, across four studies, summarised in table 3. In addition, the NEWS-FiO_2_ model^9^ was externally validated in Clarke *et al*^10^ and Forster *et al*.^26^ The NEWS+oxygen flow (3-day model) was externally validated in seven different cohorts within the same study.^24^

**Table 3:** Part 2, summary of included studies

Study	Cohort(s)	Oxygen model(s)	Comparator binary oxygen model(s)	Inclusion criteria	Admissions, n	Observations, n	Events, n	Primary outcome
Carr *et al*^24^	University Hospitals Southampton, University Hospitals Bristol and Weston NHS Foundation Trust, University College Hospital London, Wuhan, University Hospitals Birmingham, Oslo University Hospital, Guy’s and St Thomas’ Hospital	NEWS2+oxygen flow rate (3-day model)	NEWS2	COVID-19 positive, symptomatic for COVID-19	6237	6237	804	ICU admission, death
Malycha *et al*^9^	Portsmouth (all), Portsmouth (oxygen only)	NEWS-FiO_2_	NEWS	Hospital stay≥24 hours	300 672	6 600 228	13 211	Unplanned ICU admission, in-hospital death
Forster *et al*^26^	Nottingham Respiratory Patients	Additive NEWS-FiO_2_, NEWS-FiO_2_	Additive score (NEWS2+max NEWS2 in preceding 24 hours), NEWS2	Adult patients admitted to and discharged from respiratory medicine	8485	242 088	470	Death
Clarke *et al*^10^	Guy’s and St Thomas’ Influenza and COVID-19	NEWS-FiO_2_ (predicted), NEWS-FiO_2_(Bateman)	NEWS2, NEWS2	COVID-19 or influenza	3704	133 349	493	Peri-arrest, cardiac arrest, unplanned ICU admission, death

FiO_2_, fraction of inspired oxygen; ICU, intensive care unit; NEWS2, National Early Warning Score 2.

The median sample size was 7361 patient admissions. Three out of four studies used a composite primary outcome, including some or all of: cardiac/peri-arrest, unplanned ICU admission and death. The median percentage of admissions experiencing the primary outcome was 9.2% (range 4.4%–13.3%).

For each cohort, AUROC and 95% CI (where reported) for each graded oxygen model and their respective comparator binary model are presented in online supplemental table F4.

#### Quality assessment for model validation

We assessed three out of four studies at high risk of bias according to the PROBAST validation checklist.^20^ As in model development, this was most commonly due to a ‘high’ risk of bias in the analysis domain, here because of complete case analysis and/or failure to report important performance measures such as calibration curves. The NEWS2+oxygen flow rate model was considered as low risk of bias.^24^ The number of studies at risk of bias in each individual domain is highlighted in table 4. Risk-of-bias assessments for each study are found in online supplemental table F5.

**Table 4:** Part 2, risk of bias from model validation assessment

Rating	Participants	Predictors	Outcome	Analysis	Overall
High	1	0	0	3	3
Low	3	4	4	1	1

#### Results of meta-analysis

**Figure 2:**
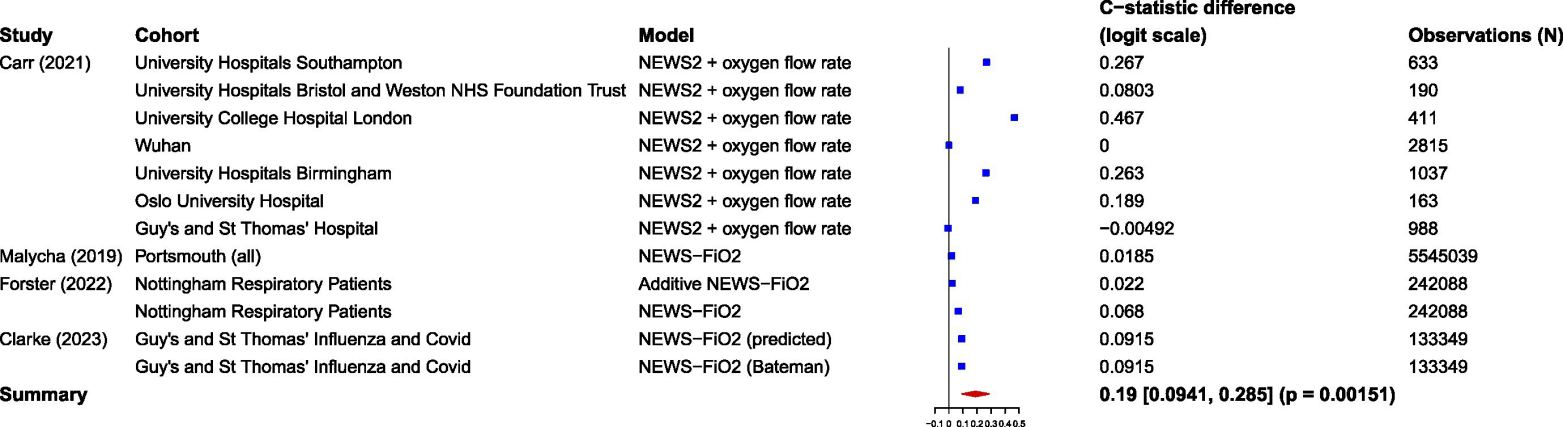
Meta-analysis of the difference in logit(AUROC) between oxygen graded and binary oxygen models. AUROC, area under the receiver operating characteristic; FiO_2_, fraction of inspired oxygen; NEWS, National Early Warning Score.

We performed random-effects meta-analysis on the difference in model performance between oxygen graded models (AUROC range 0.704–0.898) and their binary oxygen comparator model (0.687–0.900). The logit(AUROC) was on average 0.19 higher for graded oxygen models than binary oxygen models (95% CI 0.095 to 0.285, p=0.002) (figure 2).

#### Results of sensitivity analyses

We performed three sensitivity analyses:

1. Analysis in a respiratory or oxygen-dependent population (online supplemental appendix G1).
2. In models using NEWS or NEWS2 as their comparator model (online supplemental appendix G2).
3. Using study weighting according to patient admissions (rather than number of observations) (online supplemental appendix G3).

In all instances, the change to the difference in logit(AUROC) was minimal. All remained significant.

## Discussion

Our systematic review brings together the current knowledge base of graded oxygen weighting within EWS, including 16 models. We have demonstrated that oxygen classification and weighting vary considerably between models, and methodological limitations were common to both score development and validation. In addition, we believe that this is the first systematic review to demonstrate that graded oxygen weighting beyond binary weighting significantly improves EWS performance—an improvement that remained in all sensitivity analyses.

### Results in context

There are multiple examples of binary oxygen EWS in common use internationally, such as the Irish National EWS,^6^ the Aotearoa New Zealand EWS,^7^ the Cape Town Modified EWS^36^ and NEWS/NEWS2.^3^ NEWS has been validated in Colombia,^37^ Uganda^38^ and Sweden,^39^ as a few examples. NEWS was developed with knowledge of the VitalPAC EWS (ViEWS).^40 41^ When ViEWS was developed, the authors reported exploring the impact of FiO_2_ but ultimately selected a binary approach to oxygen therapy.^40^

In recent years, there have been numerous calls to improve oxygen therapy in EWS due to concerns that rising oxygen requirements are not reflected in a static score,^911^,^15^ concerns which are supported by studies demonstrating an increasing FiO_2_ before other vital sign derangements.^11 42^ For example, Prower *et al* demonstrated that FiO_2_ was the first observation to change, around 12 hours before deterioration, followed by respiratory rate at 5 hours before deterioration.^42^ The findings from our meta-analysis support this change, by showing significant improvement in discrimination with graded oxygen EWS, robust across a series of sensitivity analyses.

Although our results suggest that performance could be improved with the expansion of oxygen therapy in an EWS, the exact way this would be achieved, and its impact on ward users’ experience remains to be seen. An important consideration is that increasing the complexity of oxygen scoring may result in more transcription errors, common on paper-based charts.^43^ Therefore, the way in which weighting oxygen therapy is included in future early warning scores needs careful consideration.

We describe how 16 oxygen-graded EWS were developed. Three approaches were used to quantify oxygen: FiO_2_, flow rate or delivery device. Each has its own advantages and disadvantages.

#### Fraction of inspired oxygen

FiO_2_ is a simple scale to understand, ranging from 0.21 to 1.00. It is easy to interpret the magnitude and rate of change of an oxygen requirement. However, flow rate-based devices such as nasal cannula make up to 83% of oxygen prescriptions^9^ and therefore require conversion to FiO_2_. This adds complexity and increases the likelihood of calculation errors, especially on paper charts.^43^

The most accurate way to convert flow rate into FiO_2_ is unclear. We identified three approaches: the Bateman equation,^32^ the ‘conventional’ rule^33^ and the Wettstein calculations.^34^ Each produces subtly different estimates of FiO_2_, which impacts overall performance.^10^

In summary, if future EWS used FiO_2_, then the benefits would be a transparent and understandable scale. The drawbacks include complexity arising from conversion, and the need to establish consensus over the best method of FiO_2_ estimation.

#### Flow rate

The use of flow rate^24 25 27^ removes the need for conversion and hence conversion errors. However, flow rate is related to the delivery device,^33^ and relying on the former alone may risk deterioration going unrecognised.

#### Device

The Leeds EWS graded oxygen therapy according to delivery device.^29^ Although this is a quick and simple approach, it does not quantify oxygen delivered via the device. It may also not distinguish between patients on domiciliary/nocturnal NIV or CPAP, potentially leading to false positives and reduced sensitivity.

#### SF ratio

The SF ratio was used both by two models in this review (novelty score,^11^ MEWS_SF)^28^ and in models outside the remit of this review.^44 45^ The S/F ratio has merit in that it reflects the dependency of SpO_2_ on FiO_2_ and is therefore reactive to desaturation or rising oxygen requirement.^35^ Several studies have compared the S/F ratio to other early warning scores, often showing superior predictive performance.^45^,^47^ EWS could use the SF ratio (instead of SpO_2_ and FiO_2_ separately), if the calculation of the ratio could be automated.

There are important examples of oxygen graded EWS that did not meet our inclusion criteria. For example, the ROX index, which was developed and validated within ICU to predict mechanical ventilation in patients with pneumonia requiring HFNO^48^ and has been extensively validated across a range of settings.^49^,^52^ ROX outperformed NEWS2 at predicting deterioration in a COVID-19 cohort and triggered 4 hours earlier than NEWS2.^42^ Some obstetric EWS use FiO_2_.^53 54^ Similarly, EWS which include non-vital sign parameters and graded oxygen weighting have been proposed.^55 56^ These examples all handle oxygen in similar methods to those identified in this review. However, Youssef *et al* used other formulae to estimate FiO_2_ based on delivery device,^55^ emphasising the lack of consensus on how to accurately estimate FiO_2_.

### Strengths, limitations and considerations

There are several strengths of this review. We followed the Cochrane prognosis methods group guidance,^57^ including a preregistered protocol, adherence to quality reporting guidelines,^18^ use of a validated quality assessment tool^20^ and data extraction items directed by the Critical Appraisal and Data extraction for Systematic Reviews of Prediction Modelling Studies checklist.^19^ We also followed established methods to meta-analyse the difference between model performance.^22^

However, our results should be considered in the context of some limitations. Most included studies had significant methodological weaknesses, which were often similar to those we identified in a prior systematic review of early warning scores (although not exploring the use of graded oxygen weighting).^1^ Of the 16 graded oxygen models in this review, 15 were rated at high risk of bias commonly because they discarded missing data (13/16). Complete case analysis is a common methodological problem in EWS research^1^ and can exaggerate the observed relationship between predictor and outcome.^58^ In addition, only 7/16 models underwent any form of internal validation procedure and instead reported apparent performance on the development dataset, which is likely to be optimistic.^59^ We recommend that the development of graded oxygen EWS use imputation to handle missing data and perform internal validation using bootstrapping or cross-validation resampling techniques.^1^ Both recommendations are in accordance with Transparent Reporting of a multivariable prediction model for Individual Prognosis or Diagnosis guidance.^60^ Finally, 12/16 models were published within the last 5 years, perhaps due to COVID-19 in highlighting the limitations of binary oxygen scoring within EWS.^11^ Although we have not formally assessed publication bias, the pandemic may have influenced research priorities and publication practices, potentially introducing bias into our review.

There are some important considerations when interpreting the results of the meta-analysis. Of the 16 developed models, only 4 were eligible for part 2, as few studies focused solely on the oxygen component of their EWS. Most of these studies were at high risk of bias, again due to complete case analysis. Next, the logit(AUROC) difference should not be interpreted as the absolute improvement in AUROC for graded oxygen. A logit(AUROC) of 0.19 can be interpreted as the overall direction of change (improvement in graded oxygen), but not its magnitude. To aid interpretation, we saw AUROCs ranging from 0.70 to 0.90 (online supplemental table F4). The logit(AUROC) of 0.19 approximates to an improvement in AUROC of 4% at the lower end of the range and 1.65% at the higher end of the range. Crucially, it should be remembered that improvements in AUROC are neither necessary nor sufficient to translate to clinical benefit. Other key performance metrics, such as sensitivity and specificity, were inconsistently reported in part 2 studies, preventing further analysis that could provide a more nuanced understanding of graded oxygen model performance. Further work, including decision curve and net benefit analysis, would be required to evaluate its clinical value.^61^ Finally, most models were validated in respiratory cohorts, which may limit generalisability to an unselected hospital population. For example, respiratory patients may be more likely to be hypoxic, and any episodes of deterioration are perhaps more likely to present as worsening hypoxia, and hence the performance of oxygen graded models will appear more favourable. Model performance may also differ in a cohort with a higher event rate, as seen in studies in part 2. However, a significant performance gain was noted in one of the largest studies in the meta-analysis, in an unselected cohort.^9^

### Future work

Future work should build on the findings of this review, by using recommended methodology (such as multiple imputation and bootstrapping), to develop an EWS with the ‘best’ method for grading oxygen. Such a model will need robust validation in diverse populations, to undergo net benefit and decision curve approaches before clinical impact assessment, along with qualitative evaluation of the impact of increased score complexity on ward users.

## Conclusion

This meta-analysis provides objective evidence that increasing the complexity of oxygen therapy grading beyond binary scoring improves EWS performance. We describe 16 oxygen-graded EWS, highlighting the range of approaches to quantifying and weighting oxygen therapy in these models, and the biases introduced during development. Further work is needed to determine how the best performing methods of oxygen classification can be integrated into EWS, both in terms of recognition of deterioration and end-user functionality. However, it appears clear that including graded oxygen weighting should be considered in future iterations of EWS in clinical use.

## Supplementary material

10.1136/thorax-2024-222663 online supplemental file 1

10.1136/thorax-2024-222663 online supplemental file 2

## Data Availability

Data are available on reasonable request. All data relevant to the study are included in the article or uploaded as online supplemental information.
