# Supplementary material for: Oxygen therapy in early warning scores: a systematic review and meta-analysis
Source: Thorax. 2025 May 13;80(10):e222663. doi: 10.1136/thorax-2024-222663 (PMC12505061; doi:10.1136/thorax-2024-222663)

Oxygen therapy in early warning scores: a systematic review and meta-analysis

Supplementary appendix

Contents

Appendix A: PRISMA (abstract) checklist 2

Appendix B: PRISMA checklist 4

Appendix C: Eligibility criteria 10

Appendix D: Search strategy 11

Appendix E: Items for data extraction 12

Appendix F: Supplementary tables 13

Appendix G: Sensitivity meta-analyses 23

Appendix A: PRISMA (abstract) checklist

PRISMA 2020 for Abstracts Checklist

Section and Topic	Item #	Checklist item	Reported (Yes/No)
**TITLE**			
Title	1	Identify the report as a systematic review.	Yes - p3
**BACKGROUND**			
Objectives	2	Provide an explicit statement of the main objective(s) or question(s) the review addresses.	Yes- p3
**METHODS**			
Eligibility criteria	3	Specify the inclusion and exclusion criteria for the review.	Yes- p3
Information sources	4	Specify the information sources (e.g. databases, registers) used to identify studies and the date when each was last searched.	Yes- p3
Risk of bias	5	Specify the methods used to assess risk of bias in the included studies.	Yes- p3
Synthesis of results	6	Specify the methods used to present and synthesise results.	Yes- p3
**RESULTS**			
Included studies	7	Give the total number of included studies and participants and summarise relevant characteristics of studies.	Yes- p3
Synthesis of results	8	Present results for main outcomes, preferably indicating the number of included studies and participants for each. If meta-analysis was done, report the summary estimate and confidence/credible interval. If comparing groups, indicate the direction of the effect (i.e. which group is favoured).	Yes- p3
**DISCUSSION**			
Limitations of evidence	9	Provide a brief summary of the limitations of the evidence included in the review (e.g. study risk of bias, inconsistency and imprecision).	Yes- p3
Interpretation	10	Provide a general interpretation of the results and important implications.	Yes- p3
**OTHER**			
Funding	11	Specify the primary source of funding for the review.	n/a
Registration	12	Provide the register name and registration number.	Yes- p3

From: Page MJ, McKenzie JE, Bossuyt PM, Boutron I, Hoffmann TC, Mulrow CD, et al. The PRISMA 2020 statement: an updated guideline for reporting

systematic reviews. BMJ 2021;372:n71. doi: 10.1136/bmj.n71

Appendix B: PRISMA checklist

Reporting checklist for systematic review (with or without a meta-analysis).

Based on the PRISMA guidelines(17), downloaded from:
https://www.goodreports.org/reporting-checklists/prisma/

		Reporting Item	Page Number
**Title**			
Title	#1	Identify the report as a systematic review	Title page – pg 1
**Abstract**			
Abstract	#2	Report an abstract addressing each item in the PRISMA 2020 for Abstracts checklist	See appendix A
**Introduction**			
Background/rationale	#3	Describe the rationale for the review in the context of existing knowledge	Introduction – pg 4
Objectives	#4	Provide an explicit statement of the objective(s) or question(s) the review addresses	Intro > Objectives -pg 4
**Methods**			
Eligibility criteria	#5	Specify the inclusion and exclusion criteria for the review and how studies were grouped for the syntheses	Methods > Eligibility criteria – pg 5
Information sources	#6	Specify all databases, registers, websites, organisations, reference lists, and other sources searched or consulted to identify studies. Specify the date when each source was last searched or consulted	Methods > Search strategy – pg 6
Search strategy	#7	Present the full search strategies for all databases, registers, and websites, including any filters and limits used	Appendix D > Search strategy – pg 11
Selection process	#8	Specify the methods used to decide whether a study met the inclusion criteria of the review, including how many reviewers screened each record and each report retrieved, whether they worked independently, and, if applicable, details of automation tools used in the process	Methods > Study selection and data extraction - pg 6 Appendix C > schematic of

			inclusion criteria – pg 10
Data collection process	#9	Specify the methods used to collect data from reports, including how many reviewers collected data from each report, whether they worked independently, any processes for obtaining or confirming data from study investigators, and, if applicable, details of automation tools used in the process	Methods > Study selection and data extraction – pg 6
Data items	#10a	List and define all outcomes for which data were sought. Specify whether all results that were compatible with each outcome domain in each study were sought (for example, for all measures, time points, analyses), and, if not, the methods used to decide which results to collect	Methods > Study selection and data extraction – pg 6<br><br>Appendix E > items for data extraction – pg12.
Data items	#10b	List and define all other variables for which data were sought (such as participant and intervention characteristics, funding sources). Describe any assumptions made about any missing or unclear information	Appendix E > items for data extraction – pg 12 .
Study risk of bias assessment	#11	Specify the methods used to assess risk of bias in the included studies, including details of the tool(s) used, how many reviewers assessed each study and whether they worked independently, and, if applicable, details of automation tools used in the process	Methods > Risk of bias assessment – pg 6
Effect measures	#12	Specify for each outcome the effect measure(s) (such as risk ratio, mean difference) used in the synthesis or presentation of results	Methods > analysis – pg 7
Synthesis methods	#13a	Describe the processes used to decide which studies were eligible for each synthesis (such as tabulating the study intervention characteristics and comparing against the planned groups for each synthesis (item #5))	Methods > analysis – pg 7
Synthesis methods	#13b	Describe any methods required to prepare the data for presentation or synthesis, such as handling of missing summary statistics or data conversions	Methods > analysis – pg 7
Synthesis methods	#13c	Describe any methods used to tabulate or visually display results of individual studies and syntheses	Methods > analysis – pg 7

Synthesis methods	#13d	Describe any methods used to synthesise results and provide a rationale for the choice(s). If meta-analysis was performed, describe the model(s), method(s) to identify the presence and extent of statistical heterogeneity, and software package(s) used	Methods > analysis – pg 7
Synthesis methods	#13e	Describe any methods used to explore possible causes of heterogeneity among study results (such as subgroup analysis, meta-regression)	Methods > analysis – pg 7
Synthesis methods	#13f	Describe any sensitivity analyses conducted to assess robustness of the synthesised results	Methods > analysis – pg 7
Reporting bias assessment	#14	Describe any methods used to assess risk of bias due to missing results in a synthesis (arising from reporting biases)	n/a
Certainty assessment	#15	Describe any methods used to assess certainty (or confidence) in the body of evidence for an outcome	n/a
**Results**			
Study selection	#16a	Describe the results of the search and selection process, from the number of records identified in the search to the number of studies included in the review, ideally using a flow diagram (http://www.prisma-statement.org/PRISMAStatement/FlowDiagram)	Results > first paragraph – pg 8<br>Results > figure 1 (PRISMA flow diagram)
Study selection	#16b	Cite studies that might appear to meet the inclusion criteria, but which were excluded, and explain why they were excluded	n/a
Study characteristics	#17	Cite each included study and present its characteristics	Results > part 1 – pg 8<br>Results > part 2 > table 3 pg 13-14<br>Appendix F > table F1 and F2. Part 1, table 1 – pg 13
Risk of bias in studies	#18	Present assessments of risk of bias for each included study	Results > table 1, table 4. (overall)

			Appendix F > table F3 (pg 20) and F5 (pg 22) (individual studies)
Results of individual studies	#19	For all outcomes, present for each study (a) summary statistics for each group (where appropriate) and (b) an effect estimate and its precision (such as confidence/credible interval), ideally using structured tables or plots	Appendix F > table F4 (pg 21)
Results of syntheses	#20a	For each synthesis, briefly summarise the characteristics and risk of bias among contributing studies	Results > part 2 (pg 14)
Results of syntheses	#20b	Present results of all statistical syntheses conducted. If meta-analysis was done, present for each the summary estimate and its precision (such as confidence/credible interval) and measures of statistical heterogeneity. If comparing groups, describe the direction of the effect	Results > part 2 > meta-analysis (pg 14)
Results of syntheses	#20c	Present results of all investigations of possible causes of heterogeneity among study results	Results > part 2 > sensitivity analyses (pg 15)<br><br>Appendix G > figures G1-3 (pg 22-25)
Results of syntheses	#20d	Present results of all sensitivity analyses conducted to assess the robustness of the synthesised results	Results > part 2 > sensitivity analyses (pg 15)<br><br>Appendix G > figures G1-3 (pg 22-25)
Risk of reporting biases in syntheses	#21	Present assessments of risk of bias due to missing results (arising from reporting biases) for each synthesis assessed	n/a
Certainty of evidence	#22	Present assessments of certainty (or confidence) in the body of evidence for each outcome assessed	n/a

Discussion

Results in context	#23a	Provide a general interpretation of the results in the context of other evidence	Discussion > results in context (pg 16)
Limitations of included studies	#23b	Discuss any limitations of the evidence included in the review	Discussion > strengths, limitations and considerations (pg 18)
Limitations of the review methods	#23c	Discuss any limitations of the review processes used	Discussion > strengths, limitations and considerations (pg 18)
Implications	#23d	Discuss implications of the results for practice, policy, and future research	Discussion and Conclusions (pg 16-19)
**Other information**			
Registration and protocol	#24a	Provide registration information for the review, including register name and registration number, or state that the review was not registered	PROSPERO ID: CRD42024443362
Registration and protocol	#24b	Indicate where the review protocol can be accessed, or state that a protocol was not prepared	PROSPERO ID: CRD42024443362
Registration and protocol	#24c	Describe and explain any amendments to information provided at registration or in the protocol	See protocol
Support	#25	Describe sources of financial or non-financial support for the review, and the role of the funders or sponsors in the review	Funding statements (pg 22)
Competing interests	#26	Declare any competing interests of review authors	Declarations (pg 22)
Availability of data, code, and other materials	#27	Report which of the following are publicly available and where they can be found: template data collection forms; data extracted from included studies; data used for all analyses; analytic code; any other materials used in the review	n/a

None The PRISMA checklist is distributed under the terms of the Creative Commons Attribution License CC-BY. This checklist can be completed online using https://www.goodreports.org/, a tool made by the EQUATOR Network in collaboration with Penelope.ai

Appendix C: Eligibility criteria

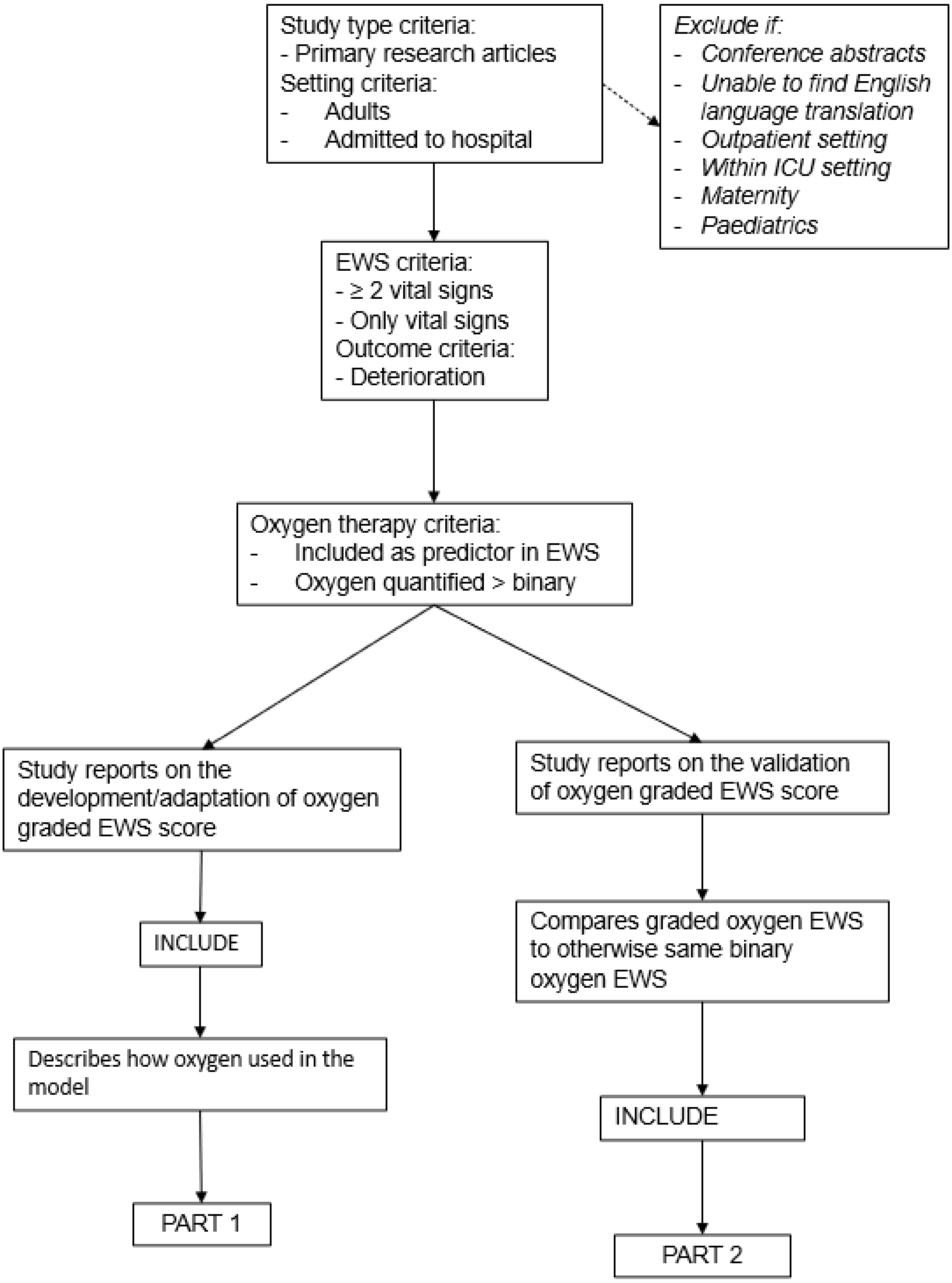

Appendix D: Search strategy

https://ovidsp.ovid.com/ovidweb.cgi?T=JS&NEWS=N&PAGE=main&SHAREDSEARCHID=19Z47FqAi490KJTy5n1HMFhvivPvyiLL0fOuGcMILmdYegXoZgbjPpQcRTqqXHNae

MEDLINE (Ovid MEDLINE® Epub Ahead of Print, In-Process & Other Non-Indexed Citations, Ovid MEDLINE® Daily and Ovid MEDLINE®) 1946 to present

1	early warning score/	381
2	(((Early Warning* or deteriorat* or Advance* or vital sign* or rapid response or dynamic) adj2 (Alert or Indicat* or Detect* or Score* or scoring or System or systems or trajectory or index or variable* or parameter*)) or ews or MEWS or R-MEWS or eCART or CEWS or TREWScore or DENWIS or DTEWS or DMEWS or POTTS or PAR-T or ViEWS-L or CART or SEWS or CREWS or News2 or news-2 or (track adj2 trigger) or trigger threshold*).ti,ab,kw.	54978
3	1 or 2	55007
4	(SpO2 or FiO2 or (Respiratory adj2 (rate or parameter* or failure)) or Oxygen* or hypox* or high flow).ti,ab,kw.	860773
5	Oxygen Inhalation Therapy/ or oxygen/	190270
6	4 or 5	922270
7	3 and 6	2459
8	7 not (Animals/ not (Animals/ and Humans/))	2238

Appendix E: Items for data extraction

Items for data extraction were taken from the CHARMS checklist(18), and included:

General study characteristics:

- Year, author, country.
- Study aims
- Study design
- Study conclusions

For part (i):

- Oxygen model:
  - Model name, non-oxygen predictors within the model
  - Methods of oxygen quantification (e.g fraction of inspired oxygen, flow rate, by delivery device), how oxygen was handled in the model (continuous, categorical, thresholds for categories and points assigned)
  - How oxygen delivery/quantity was incorporated within the model
- Model development features:
  - Method of model development
  - Outcome(s), number of outcome events in development population, time horizon
  - Internal validation, number of participants with missing data/missing data for oxygen therapy, handling of missing data
- Development cohort:
  - Setting, data source, start and end date of data collection, inclusion and exclusion criteria, sample size, type of sample size (e.g. admissions, observation sets, patients), demographics (age, sex, ethnicity)

For part (ii):

- Validation cohort:
  - Setting, data source, start and end date of data collection, inclusion and exclusion criteria, sample size, type of sample size (e.g. admissions, observation sets, patients), demographics (age, sex, ethnicity)
  - Outcome(s), number of outcome events in development population, time horizon
  - Amount and management of missing data
- Binary oxygen model:
  - Model name, included predictors, contribution to the model
- Graded oxygen model:
  - Model name, description (if study not also eligible for part 1)
- Comparison:
  - Calibration plots
  - Discrimination of both models, 95% CI or alternative, p-value of comparison
  - Any other performance metrics reported

Appendix F: Supplementary tables

Supplementary Table F1: Part 1, model development features of included studies*

Author (Year)	Oxygen model name	Data source (years)	Development method	Number of non-oxygen predictors	N (patient admissions)	N missing data (type)	Management of missing data	Internal validation performed?	Internal validation method	N (outcome events)	Primary outcome
Campbell (2020)(22)	The Queensland Adult Deterioration Detection System (Q-ADDS)	University of Chicago hospital database (2008 - 2018)	Clinical Consensus	6	224912	2069 (Patient admissions)	Discarded	No	NA	11706	ICU admission, death
Carr (2021)(23)	NEWS2 + oxygen flow rate	King's College Hospital and Princess Royal University Hospital (2020 - 2020)	Statistical Modelling	7	1276	NA (NA)	K-nearest neighbour imputation	Yes	Nested 10-fold cross validation	163	ICU admission, death
Chiu (2020)(15)	Logistic EWS	James Cook University Hospital, New Cross Hospital, Royal Papworth Hospital, University Hospitals Coventry and Warwickshire (2014 - 2017)	Statistical Modelling	6	13631	7 (Percent)	Discarded	Yes	Bootstrapping and random split	568	Unplanned ICU admission, cardiac arrest, in-hospital death
Clarke (2023)(10)	NEWS-FiO2 (predictive model)	Guy's and St Thomas' NHS Foundation Trust (2017 - 2021)	Clinical Consensus	6	3704	6020 (Observations)	Discarded	No	NA	493	Peri-arrest, cardiac arrest, unplanned ICU admission, death
Forster (2018)(24)	Nottingham EWS	Nottingham University	Clinical Consensus	6	8812	495 (Patient	Discarded	No	NA	521	In-hospital death

Author (Year)	Oxygen model name	Data source (years)	Development method	Number of non-oxygen predictors	N (patient admissions)	N missing data (type)	Management of missing data	Internal validation performed?	Internal validation method	N (outcome events)	Primary outcome
		Hospitals (2015 - 2017)				admissions)					
Forster (2022)(25)	Additive NEWS-FiO2 score	Nottingham University Hospitals (2015 - 2017)	Statistical Modelling	7	7269	218 (Patient admissions)	Discarded	Yes	Temporal validation	413	Death
Gonem (2022)(16)	Dynamic Early Warning Score (DEWS)	Nottingham University Hospitals (2015 - 2019)	Statistical Modelling	6	26470	73788 (Observations)	Discarded	Yes	Bootstrapping, 500 samples	NA	ICU admission, death
Haimovich (2020)(26)	Quick COVID-19 Severity Index (qCSI)	Not stated (2020 - 2020)	Other	2	932	Not stated (NA)	Median value imputation	Yes	Bootstrapping with 10-fold cross-validation	111	Critical respiratory illness (oxygen flow rate ≥ 10l/min, HFNO, NIV, invasive ventilation), death
Lee (2020)(27)	MEWS_SF	Asan Medical Centre (2014 - 2015)	Other	5	220	Not stated (NA)	Discarded	Yes	Temporal split validation	103	ICU admission
Malycha (2019)(9)	NEWS-FiO2	Oxford University Hospitals NHS Foundation Trust (HAVEN) (2014 - 2016)	Machine Learning	6	42764	202 (Patient admissions)	Discarded	No	NA	1669	ICU admission, death
Pimentel (2020)(11)	Novelty score	Oxford University Hospitals NHS Foundation Trust (HAVEN) (2013 - 2017)	Statistical Modelling	6	485	Not stated (NA)	Last observation carried forward Mean imputation	No	NA	62	CPAP or NIPPV/NIV initiation, ICU admission, in-hospital death
Pittard (2003)(28)	Leeds EWS	Outreach database (2001 - 2001)	Clinical Consensus	6	273	Not stated (NA)	Not stated	No	NA	NA	Unplanned ICU admission, ICU length of stay, ICU

Author (Year)	Oxygen model name	Data source (years)	Development method	Number of non-oxygen predictors	N (patient admissions)	N missing data (type)	Management of missing data	Internal validation performed?	Internal validation method	N (outcome events)	Primary outcome
											readmission, number of elective surgical cases cancelled due to lack of critical care beds, death
Tam (2017)(29)	Hamilton Early Warning Score (HEWS)	Hamilton Health Sciences Electronic Medical Records (2014 - 2014)	Clinical Consensus	6	7130	74 (Patient admissions)	Discarded	No	NA	506	Unplanned ICU admission, cardiac arrest, in-hospital death
Viglino (2020)(30)	Early Warning Score.O2 (EWS-O2)	University Hospital Grenoble-Alpes (2011 - 2018)	Clinical Consensus	3	1729	14647 (Patient admissions)	Discarded	No	NA	288	Use of NIV in ED, ICU admission, death in ED
Viglino (2020)(30)	Truncated Early Warning Score.O2 (Truncated EWS-O2)	University Hospital Grenoble-Alpes (2011 - 2018)	Clinical Consensus	2	1729	14647 (Patient admissions)	Discarded	No	NA	288	Use of NIV in ED, ICU admission, death in ED
Zhu (2020)(13)	Dynamic prediction model (DyniEWS)	James Cook University Hospital, New Cross Hospital, Royal Papworth Hospital, University Hospitals Coventry and Warwickshire (2014 - 2017)	Statistical Modelling	6	13319	Not stated (NA)	Discarded	Yes	Individualised time-series temporal split validation	4234	Unplanned ICU admission, cardiac arrest, in-hospital death

**Abbreviations: CPAP = continuous positive airway pressure, ED = emergency department, HFNO = high flow nasal oxygen, ICU = intensive care unit, NIV/NIPPV = non-invasive ventilation/non-invasive positive pressure ventilation.*

Supplementary Table F2: Part 1, cohorts included in model development studies*

Author (Year)	Oxygen model name	Setting	Inclusion Criteria	Exclusion Criteria	N (patient admissions)	Age (type)	Age spread (type)	N (male)
Campbell (2020)(22)	The Queensland Adult Deterioration Detection System (Q-ADDS)	Medical and surgical ward adult inpatients	Medical or surgical ward admissions	Lacking one full set of vital signs, or any measurement of oxygen delivery	224912	57 (Median)	41 - 69 (IQR)	94787
Carr (2021)(23)	NEWS2 + oxygen flow rate (3 day model)	Adult inpatients	COVID-19 positive, symptomatic for COVID-19	NA	1276	71.5 (Median)	57.1 - 82.6 (IQR)	742
Chiu (2020)(15)	Logistic EWS	Post-operative cardiac surgical ward	Post risk-stratified major cardiac surgery	Died intra-operatively, died in ICU before discharge to the ward, missing values	13631	NA (NA)	NA (NA)	NA
Clarke (2023)(10)	NEWS-FiO2 (predictive model)	Adult inpatients	COVID-19 positive (1st March 2020-30th March 2021) or influenza coding (1st Jan 2017 - 30th Dec 2021)	Inter-hospital transfers direct to critical care, positive COVID swab before 7 days or after 4 days from admission, incomplete observation sets	3704	61.2 (Median)	NA - NA (IQR)	1949
Forster (2018)(24)	Nottingham EWS	Respiratory Ward	Admitted onto respiratory ward	End of life coding, incomplete observation sets	8812	63.1 (Mean)	NA (NA)	3824
Forster (2022)(25)	Additive NEWS-FiO2 score	Respiratory Ward	Admitted to and discharged from respiratory ward	End of life coding, incomplete observation sets	7269	71 (Median)	61 - 81 (IQR)	3316
Gonem (2022)(16)	Dynamic Early Warning Score (DEWS)	Respiratory Ward	Admitted onto respiratory ward	First two observation sets per admission, incomplete observation sets	26470	66.3 (Mean)	17.2 (SD)	12298
Haimovich (2020)(26)	Quick COVID-19 Severity Index (qCSI)	Adult inpatients	COVID-19 positive	Oxygen requirement >6l/min within 4hrs presentation, critical illness <4hrs presentation, participant opted out of research	932	67 (Median)	55.8 - 80 (IQR)	497
Lee (2020)(27)	MEWS_SF	Haematological malignancy ward	Admitted onto haematological	DNAR before MET activation, DNAR within	220	54 (Median)	42 - 63 (IQR)	135

Author (Year)	Oxygen model name	Setting	Inclusion Criteria	Exclusion Criteria	N (patient admissions)	Age (type)	Age spread (type)	N (male)
			malignancy ward, requiring MET team activation and received treatment from the MET team	24 hours of MET contact, cardiac arrest as indication for MET call, incomplete observation set				
Malycha (2019)(9)	NEWS-FiO2	Adult inpatients	Admission >24hrs with at least one complete vital sign set	Post-ICU patients, incomplete observation sets, discharge < 24hrs	42764	64 (Mean)	19 (SD)	14887
Pimentel (2020)(11)	Novelty score	Adult inpatients	Admitted with viral pneumonia (ICD-10 coding)	No vital signs recorded outside of ICU	485	73 (Median)	57 - 84 (IQR)	224
Pittard (2003)(28)	Leeds EWS	Acute surgical ward and HDU inpatients	Admitted onto surgical ward, visited by outreach team due to: score => 3 on MEWS, cause for concern by staff, ICU stepdown	NA	273	NA (NA)	NA (NA)	NA
Tam (2017)(29)	Hamilton Early Warning Score (HEWS)	Medical and surgical ward adult inpatients	Medical or surgical ward admissions	Insufficient data to calculate HEWS score, patient admissions for palliation	7130	NA (NA)	NA (NA)	NA
Viglino (2020)(30)	Early Warning Score.O2 (EWS-O2)	Admissions from ED	ED admissions with dyspnoea	Insufficient data on RR, SpO2 and oxygen flow rate	1729	80 (Median)	66 - 87 (IQR)	841
Viglino (2020)(30)	Truncated Early Warning Score.O2 (Truncated EWS-O2)	Admissions from ED	ED admissions with dyspnoea	Insufficient data on RR, SpO2 and oxygen flow rate	1729	80 (Median)	66 - 87 (IQR)	841
Zhu (2020)(13)	Dynamic prediction model (DyniEWS)	Cardiac Surgical Wards	Post risk-stratified major cardiac surgery	Missing values due to software errors and unused oxygen delivery values, long stayers >180 days post-surgery, post re-admission to hospital, readmission to hospital following surgery, duplicates	13319	NA (NA)	NA (NA)	NA

**Abbreviations: DNAR = do not attempt resuscitation, ED = emergency department, HDU = high dependency unit, ICU = intensive care unit, ICD-10 = International Classification of Diseases (Tenth Revision), IQR = interquartile range, MET = medical emergency team, MEWS = modified early warning score, SD = standard deviation.*

Supplementary Table F3: Part 1, risk of bias assessment for individual models

Author (Year)	Oxygen model name	Domain 1: Participants	Domain 2: Predictors	Domain 3: Outcome	Domain 4: Analysis	Overall risk of bias
Campbell (2020)(22)	The Queensland Adult Deterioration Detection System (Q-ADDS)	Low	Low	Low	High	High
Carr (2021)(23)	NEWS2 + oxygen flow rate	Low	Low	Low	Low	Low
Chiu (2020)(15)	Logistic EWS	Low	Low	Low	High	High
Clarke (2023)(10)	NEWS-FiO2 (predictive model)	Low	Low	Low	High	High
Forster (2018)(24)	Nottingham EWS	High	Low	Low	High	High
Forster (2022)(25)	Additive NEWS-FiO2 score	High	Low	Low	High	High
Gonem (2022)(16)	Dynamic Early Warning Score (DEWS)	Low	Low	High	High	High
Haimovich (2020)(26)	Quick COVID-19 Severity Index (qCSI)	Low	Low	High	High	High
Lee (2020)(27)	MEWS_SF	High	Low	Unclear	High	High
Malycha (2019)(9)	NEWS-FiO2	Low	Low	Low	High	High
Pimentel (2020)(11)	Novelty score	Low	Low	Low	High	High
Pittard (2003)(28)	Leeds EWS	High	Low	High	High	High
Tam (2017)(29)	Hamilton Early Warning Score (HEWS)	High	Low	Low	High	High
Viglino (2020)(30)	Early Warning Score.O2 (EWS-O2)	Low	High	Low	High	High
Viglino (2020)(30)	Truncated Early Warning Score.O2 (Truncated EWS-O2)	Low	Low	Low	High	High
Zhu (2020)	Dynamic prediction model (DyniEWS)	Low	Low	Low	High	High

Supplementary Table F4: Part 2, performance metrics from included model comparisons

Author (Year)	Cohort	Oxygen model name	Oxygen model c-statistic (95% CI)	Comparator binary oxygen model name	Comparator model c-statistic (95% CI)
Carr (2021)(23)	University Hospitals Southampton	NEWS2 + oxygen flow rate	0.781 (not reported)	NEWS2	0.732 (not reported)
Carr (2021)(23)	University Hospitals Bristol and Weston NHS Foundation Trust	NEWS2 + oxygen flow rate	0.704 (not reported)	NEWS2	0.687 (not reported)
Carr (2021)(23)	University College Hospital London	NEWS2 + oxygen flow rate	0.831 (not reported)	NEWS2	0.755 (not reported)
Carr (2021)(23)	Wuhan	NEWS2 + oxygen flow rate	0.747 (not reported)	NEWS2	0.747 (not reported)
Carr (2021)(23)	University Hospitals Birmingham	NEWS2 + oxygen flow rate	0.815 (not reported)	NEWS2	0.772 (not reported)
Carr (2021)(23)	Oslo University Hospital	NEWS2 + oxygen flow rate	0.8 (not reported)	NEWS2	0.768 (not reported)
Carr (2021)(23)	Guy's and St Thomas' Hospital	NEWS2 + oxygen flow rate	0.716 (not reported)	NEWS2	0.717 (not reported)
Clarke (2023)(10)	Guy's and St Thomas' Influenza and Covid	NEWS-FiO2 (predicted)	0.88 (0.88-0.88)	NEWS2	0.87 (0.87-0.87)
Clarke (2023)(10)	Guy's and St Thomas' Influenza and Covid	NEWS-FiO2 (Bateman)	0.88 (0.88-0.88)	NEWS2	0.87 (0.87-0.87)
Forster (2022)(25)	Nottingham Respiratory Patients	Additive NEWS-FiO2	0.9 (0.894-0.907)	Additive NEWS2	0.898 (0.892-0.905)
Forster (2022)(25)	Nottingham Respiratory Patients	NEWS-FiO2	0.887 (0.881-0.894)	NEWS2	0.88 (0.873-0.887)
Malycha (2019)(9)	Portsmouth (all)	NEWS-FiO2	0.878 (not reported)	NEWS	0.876 (not reported)
Malycha (2019)(9)	Portsmouth (oxygen only)	NEWS-FiO2	0.823 (0.819-0.824)	NEWS	0.811 (0.809-0.814)

Supplementary Table F5: Part 2, risk of bias assessments for individual studies

Author (Year)	Domain 1: Participants	Domain 2: Predictors	Domain 3: Outcome	Domain 4: Analysis	Overall risk of bias
Carr (2021)(23)	Low	Low	Low	Low	Low
Clarke (2023)(10)	Low	Low	Low	High	High
Forster (2022)(25)	High	Low	Low	High	High
Malycha (2019)(9)	Low	Low	Low	High	High

Appendix G: Sensitivity meta-analyses

Appendix G1: Respiratory population

We anticipated that changes to quantifying oxygen therapy are likely to have the most impact amongst a respiratory or oxygen dependent cohort. We performed a subgroup analysis to compare model performance amongst this population. In effect, this meant substituting the unselected adult population data with the oxygen dependent cohort data in Malycha et al study, as all other studies used a respiratory population for validation.

Of the six oxygen graded models validated on a respiratory/oxygen dependent cohort, the logit(AUROC) was 0.19 (95% CI 0.088-0.292, p = 0.003) higher amongst the graded oxygen models than the binary oxygen models.

Figure G1: Sensitivity meta-analysis of difference in logit(AUROC) of oxygen graded vs binary oxygen models, in a respiratory/oxygen dependent population.

Study	Cohort	Model	C−statistic difference (logit scale)	Observations (N)
Carr (2021)	University Hospitals Southampton	NEWS2 + oxygen flow rate	0.267	633
	University Hospitals Bristol and Weston NHS Foundation Trust	NEWS2 + oxygen flow rate	0.0803	190
	University College Hospital London	NEWS2 + oxygen flow rate	0.467	411
	Wuhan	NEWS2 + oxygen flow rate	0	2815
	University Hospitals Birmingham	NEWS2 + oxygen flow rate	0.263	1037
	Oslo University Hospital	NEWS2 + oxygen flow rate	0.189	163
	Guy's and St Thomas' Hospital	NEWS2 + oxygen flow rate	−0.00492	988
Malycha (2019)	Portsmouth (oxygen only)	NEWS−FiO2	0.0803	1055189
Forster (2022)	Nottingham Respiratory Patients	Additive NEWS−FiO2	0.022	242088
	Nottingham Respiratory Patients	NEWS−FiO2	0.068	242088
Clarke (2023)	Guy's and St Thomas' Influenza and Covid	NEWS−FiO2 (predicted)	0.0915	133349
	Guy's and St Thomas' Influenza and Covid	NEWS−FiO2 (Bateman)	0.0915	133349
**Summary**			**0.19 [0.0882, 0.292] (p = 0.0026)**	

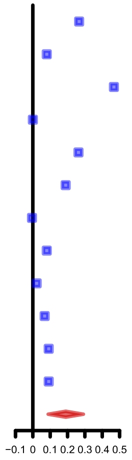

Appendix G2: NEWS/NEWS2 subgroup analysis

We performed a subgroup analysis including all studies that used NEWS or NEWS2 as their comparator model. In effect, this meant excluding the Forster et al additive-NEWS and additive NEWS-FiO2 comparisons. The difference in logit(AUROC) between graded oxygen weighting and binary oxygen models was 0.19 (95% CI 0.088-0.292, p = 0.003) amongst the five oxygen graded models compared to their equivalent NEWS/NEWS2 binary oxygen model.

Figure G2: Sensitivity meta-analysis of difference in logit(AUROC) of oxygen graded vs NEWS/NEWS2 comparator binary oxygen models.

Study	Cohort	Model	C−statistic difference (logit scale)	Observations (N)
Carr (2021)	University Hospitals Southampton	NEWS2 + oxygen flow rate	0.267	633
	University Hospitals Bristol and Weston NHS Foundation Trust	NEWS2 + oxygen flow rate	0.0803	190
	University College Hospital London	NEWS2 + oxygen flow rate	0.467	411
	Wuhan	NEWS2 + oxygen flow rate	0	2815
	University Hospitals Birmingham	NEWS2 + oxygen flow rate	0.263	1037
	Oslo University Hospital	NEWS2 + oxygen flow rate	0.189	163
	Guy's and St Thomas' Hospital	NEWS2 + oxygen flow rate	−0.00492	988
Malycha (2019)	Portsmouth (all)	NEWS−FiO2	0.0185	5545039
Forster (2022)	Nottingham Respiratory Patients	NEWS−FiO2	0.068	242088
Clarke (2023)	Guy's and St Thomas' Influenza and Covid	NEWS−FiO2 (predicted)	0.0915	133349
	Guy's and St Thomas' Influenza and Covid	NEWS−FiO2 (Bateman)	0.0915	133349
**Summary**			**0.19 [0.0882, 0.292] (p = 0.0026)**	

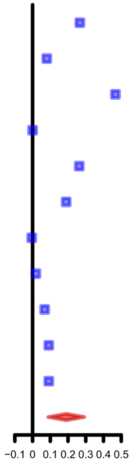

Appendix G3: Weighting by patient admissions

We performed a sensitivity analysis weighting studies according to patient admissions rather than number of observations used in the analysis. The logit(AUROC) was on average 0.175 (95% CI 0.076-0.275, p =0.004) higher for graded oxygen models versus binary oxygen models.

Figure G3: Sensitivity meta-analysis of difference in logit(AUROC) of oxygen graded vs binary oxygen models, weighted according to n(patient admissions).

**Study**	**Cohort**	**Model**	**C−statistic difference (logit scale)**	**Admissions (n)**
Carr (2021)	University Hospitals Southampton	NEWS2 + oxygen flow rate	0.267	633
	University Hospitals Bristol and Weston NHS Foundation Trust	NEWS2 + oxygen flow rate	0.0803	190
	University College Hospital London	NEWS2 + oxygen flow rate	0.467	411
	Wuhan	NEWS2 + oxygen flow rate	0	2815
	University Hospitals Birmingham	NEWS2 + oxygen flow rate	0.263	1037
	Oslo University Hospital	NEWS2 + oxygen flow rate	0.189	163
	Guy's and St Thomas' Hospital	NEWS2 + oxygen flow rate	−0.00492	988
Malycha (2019)	Portsmouth (all)	NEWS−FiO2	0.0185	217363
Forster (2022)	Nottingham Respiratory Patients	Additive NEWS−FiO2	0.022	8485
	Nottingham Respiratory Patients	NEWS−FiO2	0.068	8485
Clarke (2023)	Guy's and St Thomas' Influenza and Covid	NEWS−FiO2 (predicted)	0.0915	3704
	Guy's and St Thomas' Influenza and Covid	NEWS−FiO2 (Bateman)	0.0915	3704
**Summary**			**0.175 [0.076, 0.275] (p = 0.00358)**	

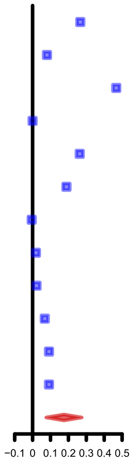

Supplement: online supplemental file 1 [file thorax-80-10-s001.pdf]
