# Supplementary material for: Oxygen therapy in early warning scores: a systematic review and meta-analysis
Source: Thorax. 2025 May 13;80(10):e222663. doi: 10.1136/thorax-2024-222663 (PMC12505061; doi:10.1136/thorax-2024-222663)
Supplement: online supplemental file 2 [file thorax-80-10-s002.pptx]

## Slide 1
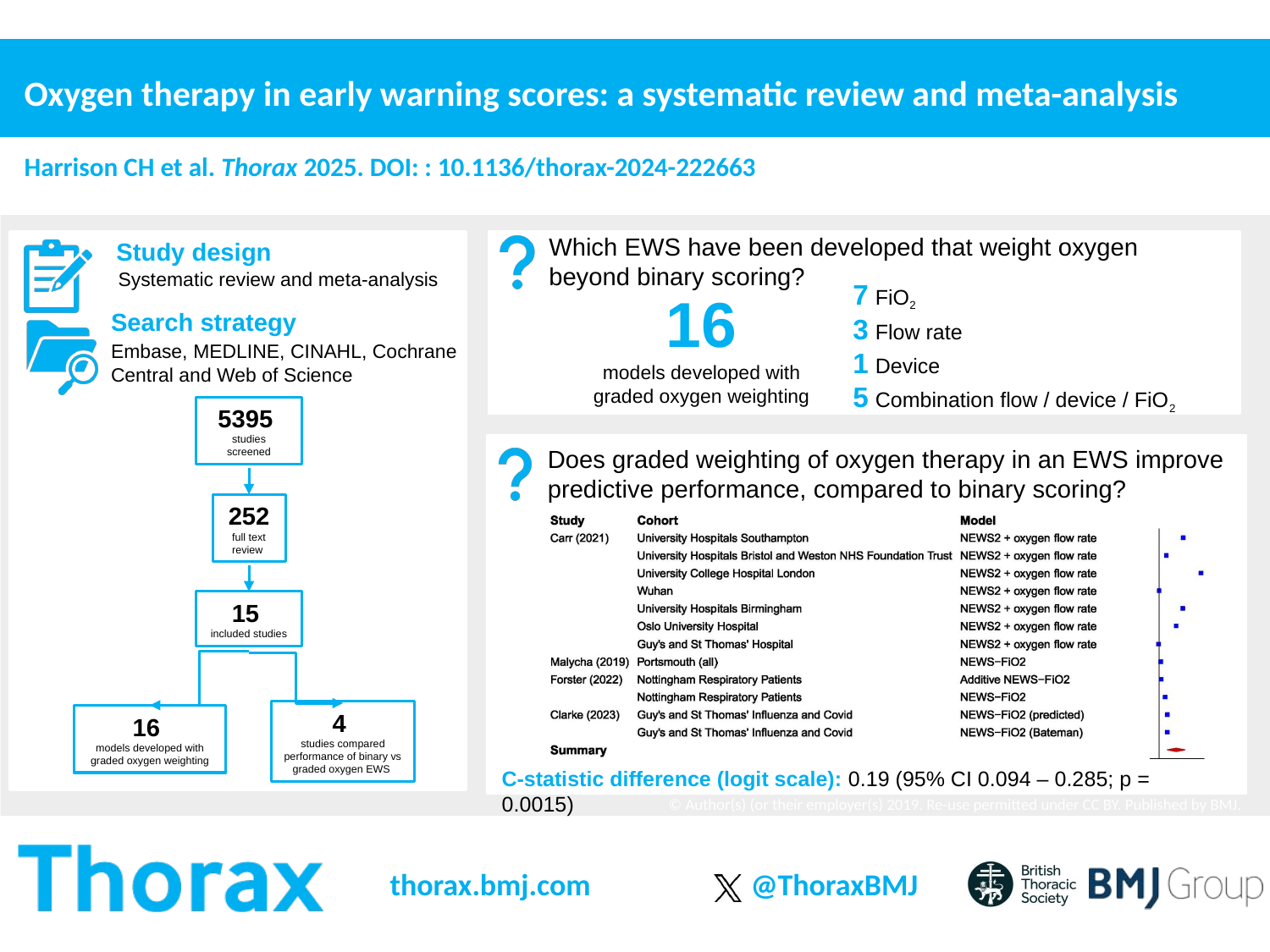

Oxygen therapy in early warning scores: a systematic review and meta-analysis
Harrison CH et al. Thorax 2025. DOI: : 10.1136/thorax-2024-222663
Which EWS have been developed that weight oxygen beyond binary scoring?
7 FiO2
3 Flow rate
1 Device
5 Combination flow / device / FiO2
16
models developed with graded oxygen weighting
Study design
Systematic review and meta-analysis
Search strategy
Embase, MEDLINE, CINAHL, Cochrane Central and Web of Science
5395
studies screened
252
full text review
15
included studies
4
studies compared performance of binary vs graded oxygen EWS
16
models developed with graded oxygen weighting
Does graded weighting of oxygen therapy in an EWS improve predictive performance, compared to binary scoring?
C-statistic difference (logit scale): 0.19 (95% CI 0.094 – 0.285; p = 0.0015)
© Author(s) (or their employer(s) 2019. Re-use permitted under CC BY. Published by BMJ.
thorax.bmj.com @ThoraxBMJ
